# Data in support of a central role of plasminogen activator inhibitor-2 polymorphism in recurrent cardiovascular disease risk in the setting of high HDL cholesterol and C-reactive protein using Bayesian network modeling

**DOI:** 10.1016/j.dib.2016.05.026

**Published:** 2016-05-21

**Authors:** James P. Corsetti, Peter Salzman, Dan Ryan, Arthur J. Moss, Wojciech Zareba, Charles E. Sparks

**Affiliations:** a Department of Pathology and Laboratory Medicine, University of Rochester School of Medicine and Dentistry, Rochester, NY, USA; b Department of Biostatistics and Computational Biology, University of Rochester School of Medicine and Dentistry, Rochester, NY, USA; c Department of Medicine – Cardiology Unit, University of Rochester School of Medicine and Dentistry, Rochester, NY, USA

**Keywords:** Recurrent cardiovascular disease risk, Pathophysiology, Plasminogen activator inhibitor-2, Bayesian network

## Abstract

Data is presented that was utilized as the basis for Bayesian network modeling of influence pathways focusing on the central role of a polymorphism of plasminogen activator inhibitor-2 (PAI-2) on recurrent cardiovascular disease risk in patients with high levels of HDL cholesterol and C-reactive protein (CRP) as a marker of inflammation, “Influences on Plasminogen Activator Inhibitor-2 Polymorphism-Associated Recurrent Cardiovascular Disease Risk in Patients with High HDL Cholesterol and Inflammation” (Corsetti et al., 2016; [1]). The data consist of occurrence of recurrent coronary events in 166 post myocardial infarction patients along with 1. clinical data on gender, race, age, and body mass index; 2. blood level data on 17 biomarkers; and 3. genotype data on 53 presumptive CVD-related single nucleotide polymorphisms. Additionally, a flow diagram of the Bayesian modeling procedure is presented along with Bayesian network subgraphs (root nodes to outcome events) utilized as the data from which PAI-2 associated influence pathways were derived (Corsetti et al., 2016; [1]).

Specifications Table

**Table:** 

Subject area	Clinical research
More specific subject area	Cardiovascular disease risk
Type of data	Text file and figures
How data was acquired	Prospective study
Data format	Raw, analyzed
Experimental factors	Determination of clinical, blood biomarker, and genetic polymorphism parameters
Experimental features	Recurrent coronary events followed in 166 post-MI patients for 26 months
Data source location	USA
Data accessibility	Data are within this article

## Value of the data

• Data on recurrent coronary events in post-MI patients having concurrently high levels of HDL-C and CRP are well-suited for studies challenging the notion of HDL as the “good cholesterol”.
• The data may be instrumental in further elucidation of the role of inflammation in the dysfunctional transformation of HDL from anti-atherogenic to pro-atherogenic.
• The multivariable data involving clinical, blood biomarker, and genetic polymorphism variables provide opportunities for the application of advanced knowledge discovery techniques in a holistic manner potentially leading to new insights on the pathophysiology of cardiovascular disease.
• The three Bayesian network subgraphs (root nodes to outcome events) represent data that for the variables in a subgraph present all intervening influence relationships some of which may be worthy of further explorations in future studies.

## 1 Data

**Fig. 1:**
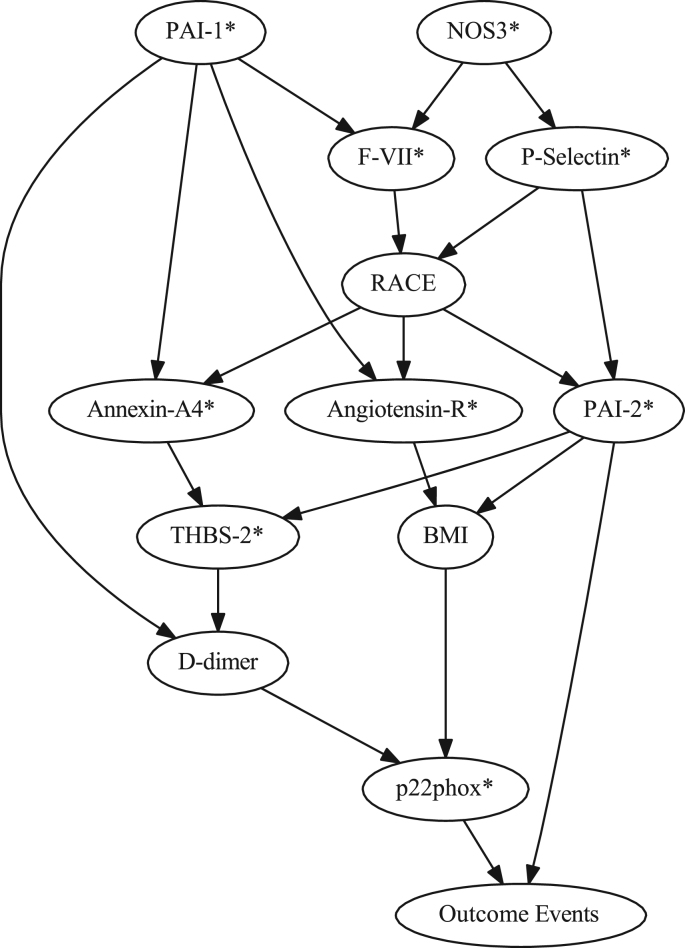
Bayesian network subgraph (root nodes to outcome events node pre-specified as a terminal node) demonstrating the PAI-2 SNP (rs6095) and the p_22_phox SNP (rs4673) as parents of outcome events as well as demonstrating further influence relationships among all contributing variables.

**Fig. 2:**
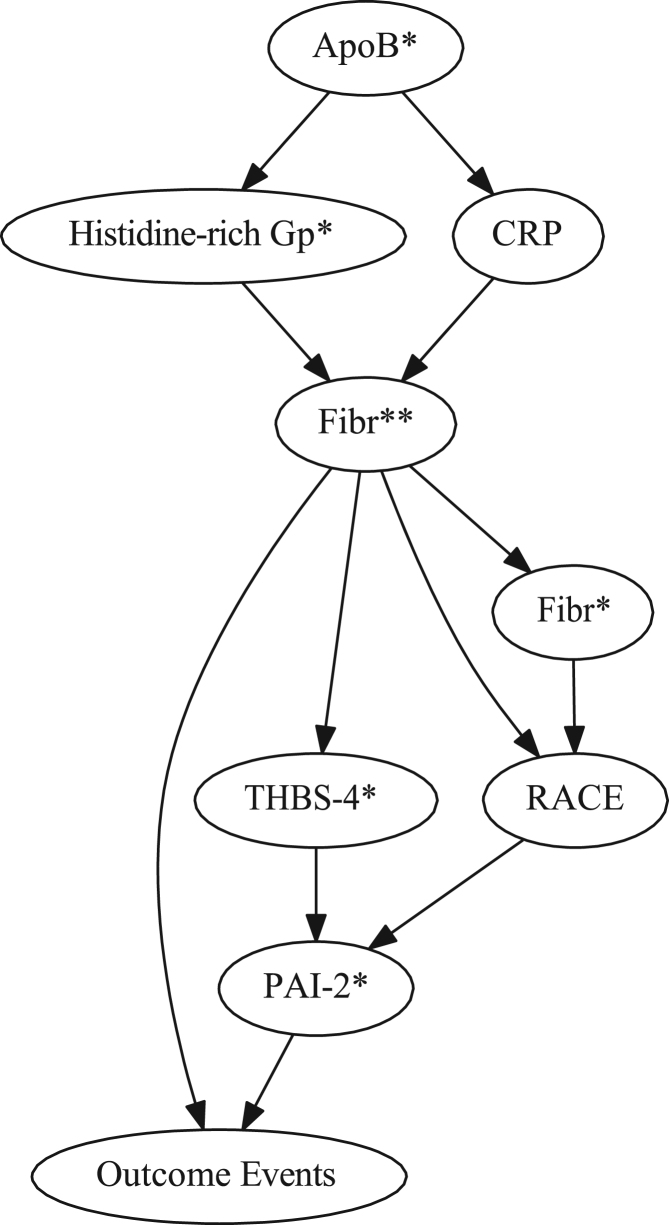
Bayesian network subgraph (root nodes to outcome events node pre-specified as a terminal node) demonstrating the PAI-2 SNP (rs6095) and the fibrinogen SNP (rs4220) as parents of outcome events as well as demonstrating influence relationships among all contributing variables.

**Fig. 3:**
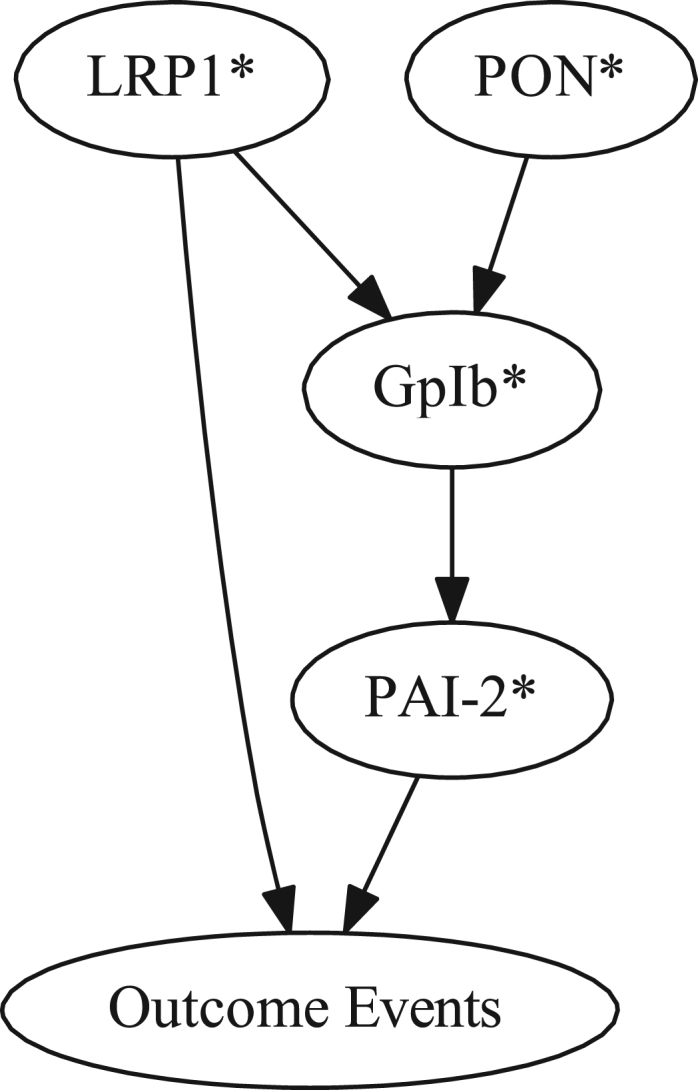
Bayesian network subgraph (root nodes to outcome events node pre-specified as a terminal node) demonstrating the PAI-2 SNP (rs6095) and the LRP1 (low-density lipoprotein receptor-related protein-1) SNP (rs1800156) as parents of outcome events as well as demonstrating influence relationships among all contributing variables.

The data (data file 1) consist of values of 4 clinical parameters (gender, age, race, BMI) along with 17 blood biomarkers and 53 SNPs all presumptively associated with cardiovascular disease risk in 166 non-diabetic post-MI patients having concurrently high levels of HDL-C and CRP. The data also include cardiovascular disease outcomes (cardiac death, MI, unstable angina) in the patients with a mean follow-up time of 26 months. Additional data (Fig. 1, Fig. 2, Fig. 3) include 3 Bayesian network subgraphs (root nodes to the node representing recurrent coronary event outcome that in each case was pre-specified as a terminal node) which delineate three parent sets of outcome that in each case include a PAI-2 SNP (rs6095) as one of the two parents of outcome.

## 2 Experimental design, materials and methods

### 2.1 Experimental design

**Fig. 4:**
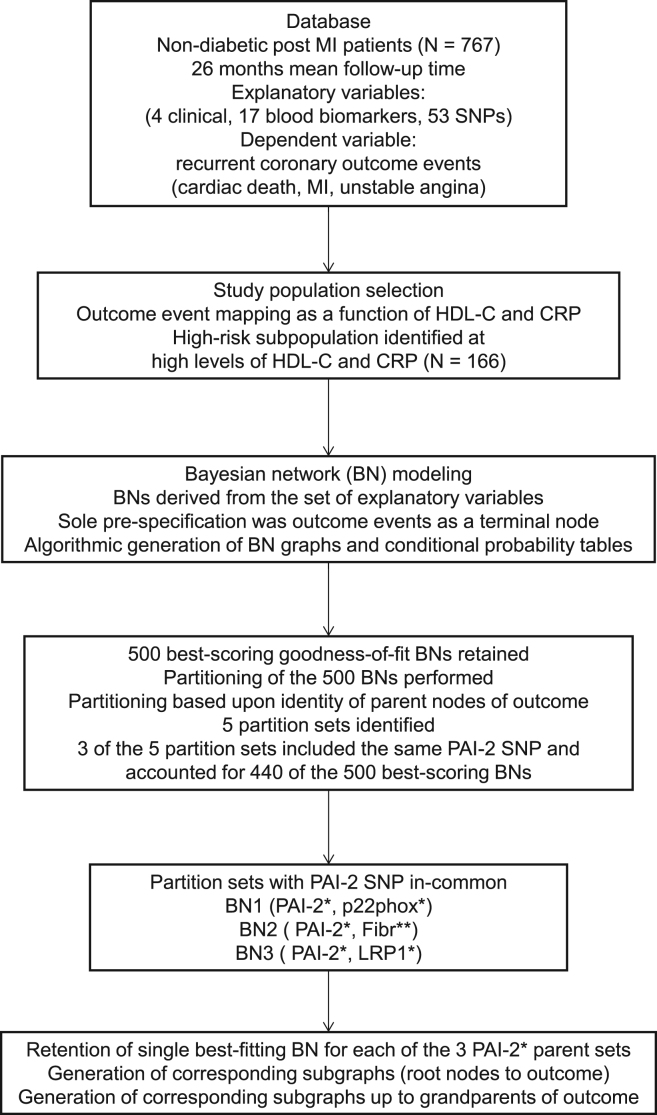
Flow diagram of Bayesian network modeling approach.

High levels of HDL-C were chosen to avoid potentially confounding effects on CVD risk by low levels of HDL-C. From the study using Bayesian network modeling, further data were derived consisting of three subgraphs (root nodes to recurrent coronary outcome events). These subgraphs delineated influence relationships among contributing variables with each of the three having in-common a PAI-2 SNP (rs6095) as a parent of outcome. A flow diagram of the generation of the data is given in Fig. 4.

### 2.2 Study population

The study population consisted of non-diabetic post-MI patients having concurrently high levels of HDL-C and CRP [2] that were drawn from the Thrombogenic Factors and Recurrent Coronary Events (THROMBO) postinfarction study [3] using outcome event mapping, a graphical approach for the identification of specific patient subgroups [4]. The THROMBO study was an investigation of blood biomarkers as predictors of risk for recurrent coronary events. Patients had blood markers determined two months after having an MI with subsequent following for an average of 26 months for recurrent coronary events (cardiac death, additional MI, unstable angina). The THROMBO study was carried out with approval of and according to guidelines of Research Subjects Review Boards of participating institutions including acquisition of written informed consent.

### 2.3 Laboratory analyses

Fasting sera and plasma were prepared two months after index MI. Apolipoprotein B (apoB), total cholesterol, lipoprotein-associated phospholipase A2 (Lp-PLA2) activity, apolipoprotein A-I (apoA-I), HDL, triglyceride, glucose, insulin, lipoprotein(a) (Lp(a)), plasminogen activator inhibitor-1 (PAI-1), von Willibrand factor antigen (vWF), fibrinogen, D-dimer, factor VII, and factor VIIa were determined as described previously [3]. CRP and serum amyloid A (SAA) were determined by immunonephelometry [5]. Genotypings of 53 SNPs presumptively associated with cardiovascular disease risk were performed on DNA samples isolated from buffy coats that were stored a *t* −70 °C until analysis using standard techniques as described previously [6], [7].

### 2.4 Bayesian network modeling

Bayesian network (BN) modeling generates a directly interpretable graphical representation of the joint probability distribution over a set of random variables, in this case, over our data comprised of four clinical, 17 blood biomarker, and 53 SNP variables along with recurrent coronary events pre-specified as a terminal node. What this means is that a network is a pictorial representation of the influences that variables have on each other. In the case at hand, it allows identification of pathways of influence that the variables have on each other in leading to recurrent coronary events. In the representation, variables appear within enclosures denoted as “nodes” and influences between variables appear as arrows pointing from a “parent” node to a “child” node. A key property of BNs for the current work is that for a terminal node (a node without any children), the direct and presumably sole causal influences on that terminal node are its parent nodes. In the present case, as stated above, recurrent coronary events were pre-specified as a terminal node and thus, identification of its parent node variables would result in identification of significant and direct influences on recurrent risk. In addition, identification of node variables and relationships in the locale of the recurrent risk node potentially could provide information more generally related to pathophysiologic mechanisms leading to risk.

Thus, a series of BNs based on the data of the current study were generated and ranked using an estimation algorithm and model selection criteria, respectively, as described in detail previously [1]. Using this approach, the 500 best-scoring BNs were retained for further consideration. The BNs were then partitioned based upon commonality of the parental nodes of outcome. Remarkably, only five partition patterns of the parental node of outcome resulted three of which demonstrated in-common a SNP of PAI-2 (rs6095) accounting for 440 of the 500 BNs, thus highlighting the key role of PAI-2 in recurrent risk. To focus on the regions of the three BNs most closely related to outcome and in view of the complexity of the full BNs, corresponding subgraph data (root nodes to outcome) were generated.
